# Matrix metalloproteinase imaging and survival prospects with aging in murine aortic aneurysm

**DOI:** 10.1186/s13550-026-01521-w

**Published:** 2026-09-22

**Authors:** Mean Ghim, Onur Varli, Azmi A. Ahmad, Afarin Neishabouri, Gunjan Kukreja, Zhengxing Zhang, Saalar Zarnegar, Nicholas Gangemi, Keshvad Hedayatyanfard, Jakub Toczek, Lingyun Wang, Jiasheng Zhang, Chi Liu, Yongjian Liu, Robert J. Gropler, Mehran M. Sadeghi

**Affiliations:** 1 Yale Cardiovascular Research Center, Section of Cardiovascular Medicine, Department of Internal Medicine, Yale School of Medicine, 300 George Street, Room 770G, New Haven, 06511 CT USA; 2 Veterans Affairs Connecticut Healthcare System, West Haven, CT USA; 3 Department of Radiology and Biomedical Imaging, Yale University, New Haven, CT USA; 4 Department of Radiology, Washington University, St. Louis, MO USA

**Keywords:** Aortic aneurysm, Aging, Matrix metalloproteinases, Molecular imaging, PET/CT

## Abstract

**Background:**

Matrix metalloproteinases (MMPs) are elevated in aortic aneurysm (AA), where older age is a risk factor. The relationship among aging, aortic MMP activity, and AA development has not been systematically evaluated. This study leveraged MMP-targeted molecular imaging to investigate how aging affects aortic MMP activity, aneurysm development, and survival.

**Results:**

AA development and animal survival were monitored for 28 days after Angiotensin (Ang)-II infusion in 8-10-week-old (young) and > 51-week-old (old) apolipoprotein E (*Apoe*)^*−/−*^ mice. Old animals’ survival at 28 days was significantly lower than that of young Ang-II-infused *Apoe*^*−/−*^ mice (*P* < 0.05). ^64^Cu-RYM2 PET/CT showed significantly higher aortic MMP activation before and 1 week after Ang-II infusion in old compared to young *Apoe*^*−/−*^ mice. The ^64^Cu-RYM2 signal was significantly higher in animals that did not survive 28 days than in those that did (*P* < 0.01). MMP activity significantly increased after Ang-II infusion and correlated with aortic macrophage infiltration. The increase in MMP activity preceded aortic dissection. Finally, MMP activity was heterogeneous along the aorta of control and Ang-II-infused young and old *Apoe*^*−/−*^ mice, and of old wild-type mice.

**Conclusions:**

Aging is associated with increased MMP activity along the aorta and worse AA survival. MMP-targeted molecular imaging provides insights regarding aneurysm survival prospects in this exploratory cohort. Combining MMP inhibitors and tracers may help prevent and track aneurysm growth, dissection, and rupture.

**Supplementary Information:**

The online version contains supplementary material available at https://doi.org/10.1186/s13550-026-01521-w.

## Background

Each year, aortic aneurysms cause between 150,000 and 200,000 deaths worldwide [1–3]. Advanced age is a major risk factor for developing abdominal aortic aneurysms (AAA), and although AAA sizes display a similar age distribution among those screened for AAA, death from rupture is rare before age 65 [4, 5]. Advanced age is also a risk factor for thoracic aortic aneurysms (TAA) [6, 7]. TAA dissection in younger patients usually occurs in those with connective tissue disorders like Marfan and Ehlers-Danlos syndromes, but dissection of the more common “degenerative” TAA is more often seen in older patients [7, 8]. The specific factors that contribute to or mediate the higher incidence of aortic aneurysms with advancing age remain unclear.

Matrix metalloproteinases (MMPs) are a multigene family of zinc-dependent endopeptidases that, once active, can degrade extracellular matrix components. MMP activity is tightly regulated at multiple levels, including gene expression, zymogen activation, and inhibition by tissue inhibitors of MMPs [9]. In aortic aneurysm tissues from patients undergoing surgery for aneurysm complications, aortic dissection and rupture, MMP expression and activity are upregulated compared to the normal aorta [10, 11]. Similarly, MMPs are upregulated in animal models of aortic aneurysms [10]. While it is likely that increased MMP expression and activity contribute to the pathogenesis of aneurysm complications, one cannot rule out that the increased MMP activity observed in such surgical specimens results from the dissection or rupture that prompts surgical intervention. The expression of some members of the MMP family is increased with aging [12, 13]. However, the relation between aging and MMP activity, and its contribution to the increased prevalence of aortic aneurysms and their complications with aging, has not been systematically evaluated.

MMP-targeted molecular imaging is a promising approach for in vivo tracking of vessel wall biology and may be valuable for aneurysm risk stratification. Recently, several positron emission tomography (PET) and single photon emission computed tomography (SPECT) MMP-targeting radiotracers, such as ^64^Cu-RYM2 and ^99m^Tc-RYM1, have been developed and shown promise for detecting aortic aneurysms in murine models [11, 14, 15]. Here, we leveraged MMP-targeted molecular imaging in combination with tissue zymography to (a) assess how aging affects murine aneurysm development and survival, (b) investigate the effect of aging on aortic MMP activity, (c) evaluate the performance of MMP-targeted molecular imaging (^64^Cu-RYM2 PET/CT) in predicting aneurysm outcome, and (d) elucidate the spatial and temporal relationship between MMP activity and aortic dissection.

## Materials and methods

### Animals

Male C57BL/6J (wild-type, WT, *n* = 11) and apolipoprotein E-deficient (*Apoe*^*−/−*^, *n* = 95) mice, originally from Jackson Laboratory, were bred in-house. A flowchart summarizing the experimental animal groups is provided in the Supplemental Material (Supplemental Fig. 1). In vivo imaging studies focused on 8-10-week-old (“young”) and > 51-week-old (“old”) mice, while tissue analysis included an additional intermediate-age, fully adult (12-17-week-old) group. The age of old mice was selected, balancing sufficient time to detect meaningful changes with the risk of waiting too long, when animals often develop skin lesions and other abnormalities that may confound the results. An additional 8-10-week-old and 8-month-old mice were used to collect blood samples for MMP activity analysis. To induce aortic aneurysm, *Apoe*^*−/−*^ mice were infused with recombinant human angiotensin II (Ang II, Santa Cruz Biotechnology) at 1,000 ng/kg/min for up to 28 days via osmotic minipumps (models 2001 or 2004; Alzet) implanted subcutaneously under isoflurane anesthesia. Postoperative analgesia was provided as a single subcutaneous dose of extended-release buprenorphine (Ethiqa XR, 3.25 mg/kg). For animals found dead, aortic rupture was confirmed as the cause of death by necropsy whenever possible. While no animal was removed from the study based on clinical data, a pre-defined group of animals was removed for tissue collection on day 3, and is not included in the survival analysis.

### Tracer synthesis and labeling

The synthesis and radiolabeling of RYM2 with ^64^Cu were performed according to previously reported procedures [11]. Briefly, cyclotron-produced ^64^CuCl₂ (Washington University School of Medicine Mallinckrodt Institute of Radiology Cyclotron Facility) was converted to ^64^Cu(OAc)₂ by adding 50 µL of 0.5 M NaOAc (pH 3.5). RYM2 (5 nmol in 5 µL DMSO) was mixed with 60 µL of 0.1 M NaOAc (pH 5.5), added to the radioisotope, and heated for 30 min at 45 °C. The radiolabeled solution was cooled to room temperature and subjected to quality control by radio high-performance liquid chromatography (HPLC). HPLC separation was performed on a Phenomenex C18 reverse-phase column (Luna 5 μm C18(2), 100 Å, LC Column 250 × 4.6 mm). Gradient elution was performed using water with 0.1% trifluoroacetic acid (TFA) as mobile phase A and acetonitrile with 0.1% TFA as mobile phase B. The flow rate was set at 1 mL/min, with a 5–95% B gradient over 10 min, followed by a 1-min isocratic phase. The gradient was then linearly returned to the initial conditions by the end of the run. Chromatograms were monitored by ultraviolet detection at 254 and 214 nm and by in-line radioactivity detection for ^64^Cu-labeled compounds. The specific activity of ^64^Cu-RYM2 was 78.2 ± 8.4 GBq/µmol. The synthesis and radiolabeling with ^99m^Tc of RYM1 (a RYM2 homolog) were as described [14].

### PET/CT imaging

^64^Cu-RYM2 PET/CT was performed as described [11]. Briefly, under inhaled isoflurane anesthesia, the animals were injected intravenously with 11.73 ± 3.50 MBq of ^64^Cu-RYM2 and 30 µL plus (body weight in grams) µL of Exitron nano 12000 (Viscover Imaging). A CT acquisition was then performed, followed by a 10-minute PET acquisition on a dedicated small-animal PET/CT scanner (Inveon PET/CT, Siemens Medical Solutions), initiated 50 min after tracer injection. CT images for attenuation correction were reconstructed at a downsampled resolution (0.815 × 0.815 × 0.796 mm) using the Feldkamp cone-beam algorithm. CT images for co-registration with PET images were reconstructed using a 3D ordered-subset expectation-maximization (OSEM3D) algorithm with an isotropic voxel size of 0.111 mm.

Emission data were reconstructed using the manufacturer’s software (Inveon Workstation, version 2.0) with the OSEM3D algorithm (2 iterations, 16 subsets), followed by a maximum a posteriori probability reconstruction (MAP; 25 iterations), yielding images with a voxel size of 0.388 × 0.388 × 0.796 mm and incorporating corrections for decay, attenuation, scatter, normalization, and randoms. PET images were normalized to the injected dose and body weight and displayed on a standardized uptake value (SUV) scale. One Ang II-infused animal in the “old” group died during imaging, and its images were excluded from analysis.

### PET/CT image analysis

Quantitative images analysis was performed using 3D Slicer software (version 5.2.2; https://www.slicer.org/) [16], and AMIDE software (version 1.0.6; https://amide.sourceforge.net/) was used to display images. Contrast-enhanced CT images were used to localize the aorta and define volumes of interest (VOIs) along the aorta as follows: the descending thoracic aorta extended from the aortic arch to the diaphragm, the suprarenal aorta from the diaphragm to the renal arteries, and the infrarenal aorta from the renal arteries to the iliac arteries. These VOIs were then applied to the PET volume, with uptake adjacent to the lumen area included when present, and the spillover signal from the kidneys, identified as a smooth extension of the kidney signal, excluded. Inter-observer variability was assessed through independent analyses of a subset of imaging data by two observers (AA, NG), one with over three years of experience. The intraclass correlation coefficient for the measurement was 0.87, confirming the reproducibility of the image analysis. A previously acquired PET/CT dataset of young *Apoe*^−/−^ mice (*n* = 5) [11] was reanalyzed for use in the current study. Image analysis of this dataset was independently repeated using the same workflow, and the reanalysis measurements closely matched the original values (intraclass correlation coefficient = 0.98).

### Zymography

Animals were anesthetized and perfused with cold phosphate-buffered saline (PBS). The aorta was quickly cleaned and excised from surrounding tissues, then cut into predefined anatomical segments and embedded in Optimal Cutting Temperature (OCT) compound. The ascending aorta (AAo), which included the aortic arch, extended from above the aortic valve to the left subclavian artery. The descending thoracic aorta (DTA) extended from the left subclavian artery to the diaphragm. The suprarenal abdominal aorta (SRA) extended from the diaphragm to the left renal artery, and the infrarenal abdominal aorta (IRA) extended from the renal arteries to the iliac bifurcation. An aorta with evidence of dissection on day 3 (intramural hematoma accumulation [17]) was excluded from analysis. Tissue MMP activity was detected and quantified in 1 µg of protein lysate, as previously described [14], using the commercial fluorometric MMP activity assay, the SensoLyte 520 Generic MMP Activity Kit from AnaSpec, following the manufacturer’s instructions. MMP activity was measured at excitation and emission wavelengths of 490 and 520 nm, respectively, using either an Agilent BioTek Synergy H1 or an Applied Biosystems 7500 fluorescence detection system. Cross-instrument normalization was performed using a correction factor derived from biologically matched reference samples. For blood cell MMP activity, whole blood was collected from young (8–10 weeks old) and old (8–9 months old) *Apoe*^*−/−*^ mice, centrifuged at 1000 x g for 15 min, and the cellular fraction was isolated, lysed, and analyzed as described above.

### Near infrared fluorescence imaging

MMPSense 645 FAST (Revvity), an activatable pan-MMP probe that fluoresces after cleavage by MMPs, was prepared according to the manufacturer’s instructions and administered intravenously to animals at a dose of 160 nmol/kg. Twenty-four hours later, the animals were anesthetized. A cold PBS solution was infused into the left ventricle, and the right atrium was incised to serve as the outflow. The aorta was quickly cleared of surrounding tissue under a stereomicroscope (MZ9.5, Leica), then removed and fixed in 4% paraformaldehyde in PBS. It was opened longitudinally, flat-mounted, and imaged as a whole-mount preparation on a DMi8 widefield fluorescence microscope (Leica) with a compatible filter set. Autofluorescence images provided anatomical reference. ImageJ (NIH) was used to quantify the mean MMPSense 645 fluorescence intensity across different regions of interest (ROIs) defined on the autofluorescence image.

### Autoradiography

Autoradiography was performed as previously described [14]. Mice were injected intravenously with ⁹⁹ᵐTc-RYM1 (26.8 ± 2.3 MBq). Two hours later, the animals were anesthetized, and the aorta was dissected free of surrounding tissues, placed on a phosphor imaging plate (MultiSensitive Phosphor Screen; PerkinElmer) with standards of known activity, and scanned with a high-resolution phosphor imager (Typhoon Trio; GE Healthcare Life Sciences). Digital images were analyzed in ImageJ (NIH) by drawing ROIs over predefined anatomical segments of the aorta. Tracer uptake was reported as decay-corrected percent injected dose per square centimeter (%ID/cm²).

### Statistical analysis

Data are presented as mean ± standard deviation (SD). Normality was assessed with the Shapiro-Wilk test before applying parametric tests. Unpaired or paired two-tailed t*-*tests were used to compare two groups. For comparisons involving more than two groups, one-way analysis of variance (ANOVA) with Tukey’s post hoc multiple-comparison test was used. Repeated-measures (RM) ANOVA was used for matched measurements. Kaplan–Meier survival curves were compared using the log-rank (Mantel–Cox) test. The statistical significance of the association between SUVmax as a dichotomous variable and survival was determined using Fisher’s exact test. The Pearson correlation test evaluated the linear relation between MMP activity and macrophage marker (CD68) expression. One outlier was excluded from this correlation analysis based on the robust regression and outlier removal test. A *P* value < 0.05 was considered statistically significant. Statistical analyses were conducted using GraphPad Prism Software, version 10.6.

## Results

### Animal age and rupture-free survival in aortic aneurysm

**Fig. 1:**
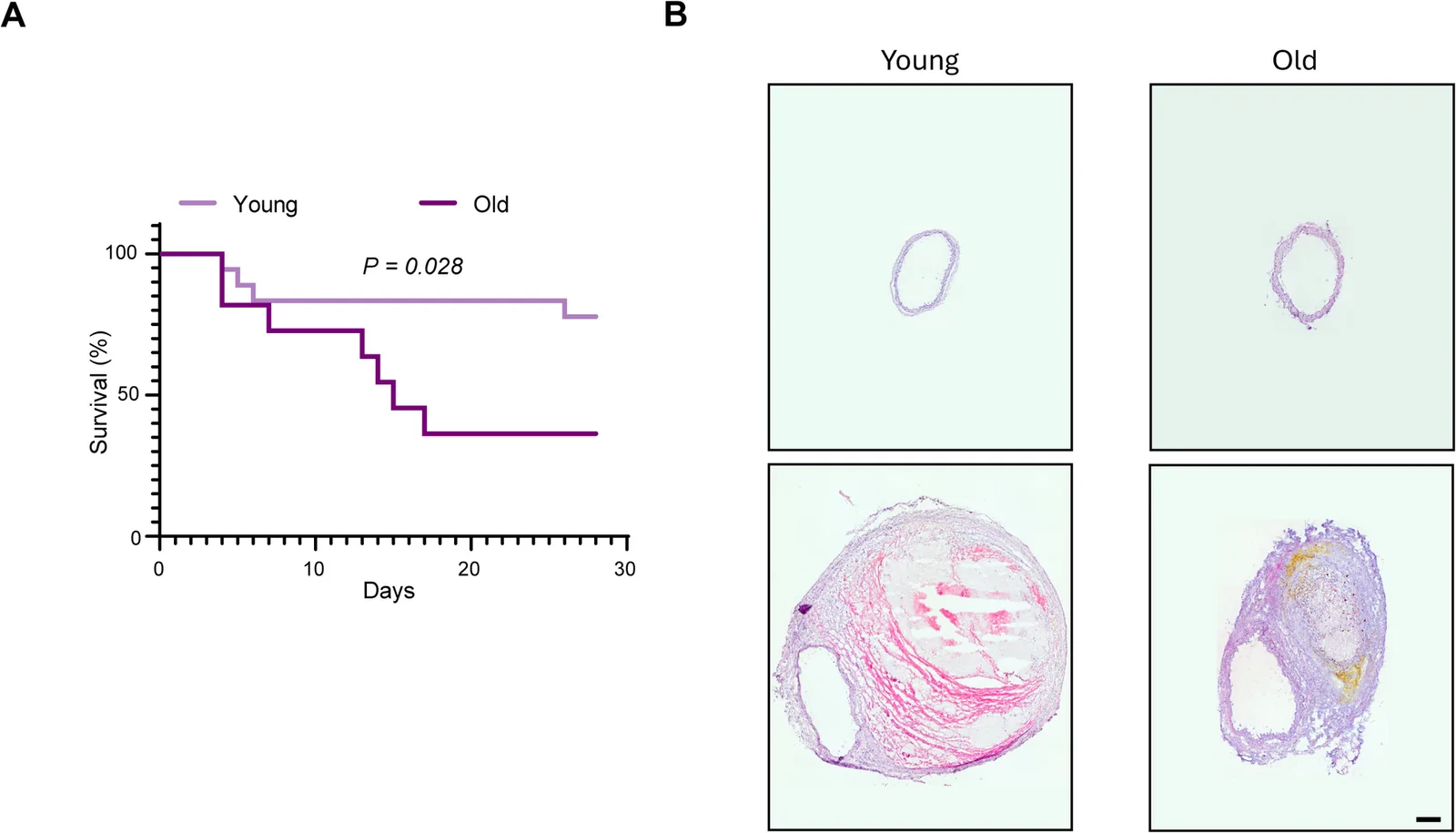
Age and survival in angiotensin II-infused *Apoe*^*−/−*^ mice. (**A**) Kaplan–Meier survival curves for young (8–10 weeks) and old (> 51 weeks) *Apoe*^*−/−*^ mice during 28 days of angiotensin II infusion (*P* < 0.05). (**B**) Illustrative examples of H&E-stained cross-sections of the suprarenal abdominal aorta from young and old mice without (top panels) and with AAA (bottom panels). Scale bar = 200 μm

Angiotensin II infusion triggers aortic aneurysm development and death from rupture in *Apoe*^*−/−*^ mice [18]. A comparison of rupture-free survival to 28 days in young (8–10 weeks, *n* = 18) and old (> 51 weeks, *n* = 11) *Apoe*^*−/−*^ mice infused with Ang II showed significantly lower survival in older animals (Fig. 1A, *P* < 0.05). Except for one old animal that was lost during imaging, all other deaths (4 in the young group and 6 in the old group) were attributable to aortic rupture. Morphometric analysis of the aorta in surviving animals showed the presence of AAA in 5 of 14 young and 1 of 4 old mice (Fig. 1B).

### Animal age, aorta MMP activation, and aneurysm outcome

**Fig. 2:**
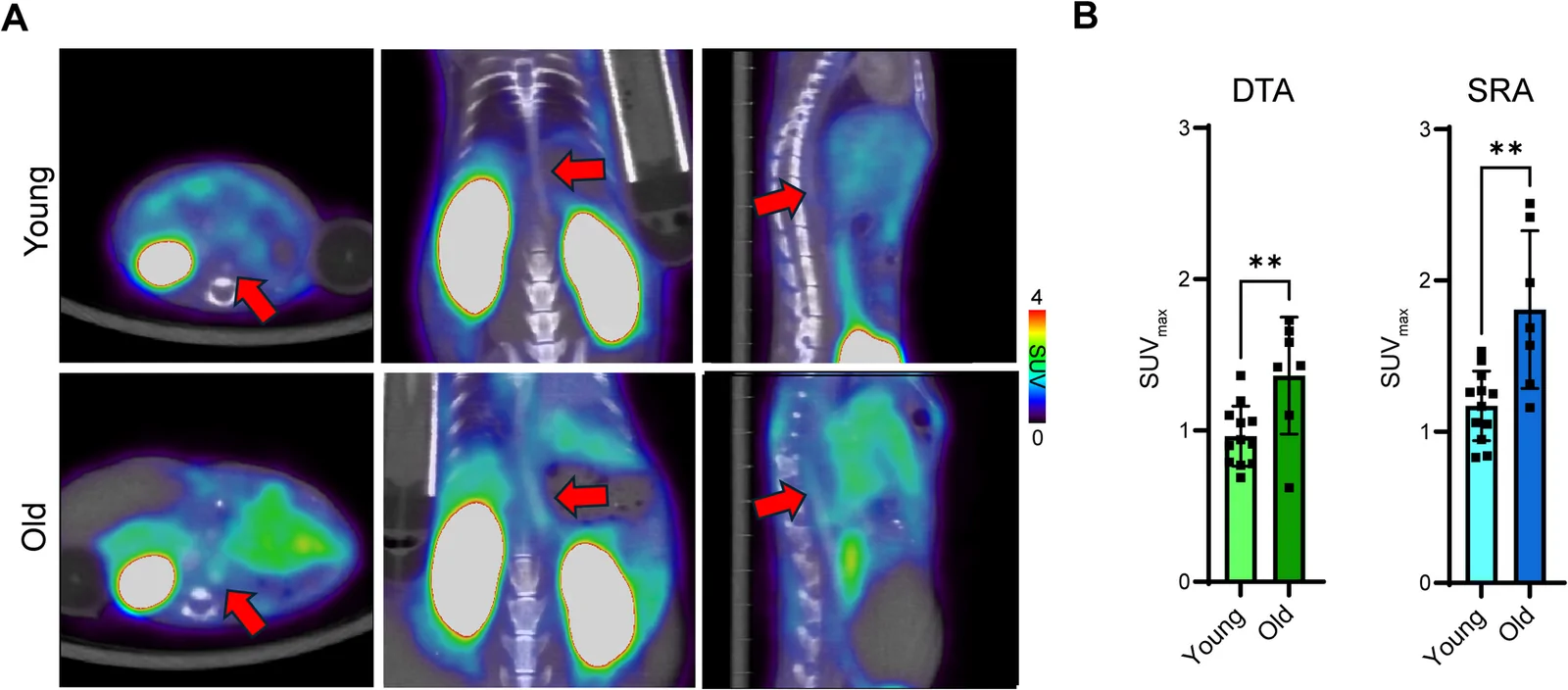
Age and ^64^Cu-RYM2 PET/CT in angiotensin II-infused *Apoe*^*−/−*^ mice. (**A**) Illustrative examples of fused ^64^Cu-RYM2 PET/contrast-enhanced CT images (axial, coronal, and sagittal) acquired 7 days after starting angiotensin II infusion in young (8–10 weeks) and old (> 51 weeks) *Apoe*^*−/−*^ mice. Red arrows indicate the suprarenal abdominal aorta. (**B**) Quantification of the ^64^Cu-RYM2 signal in the descending thoracic aorta (DTA) and suprarenal abdominal aorta (SRA) in young and old mice. **: *P* < 0.01. SUV: Standardized uptake value

**Fig. 3:**
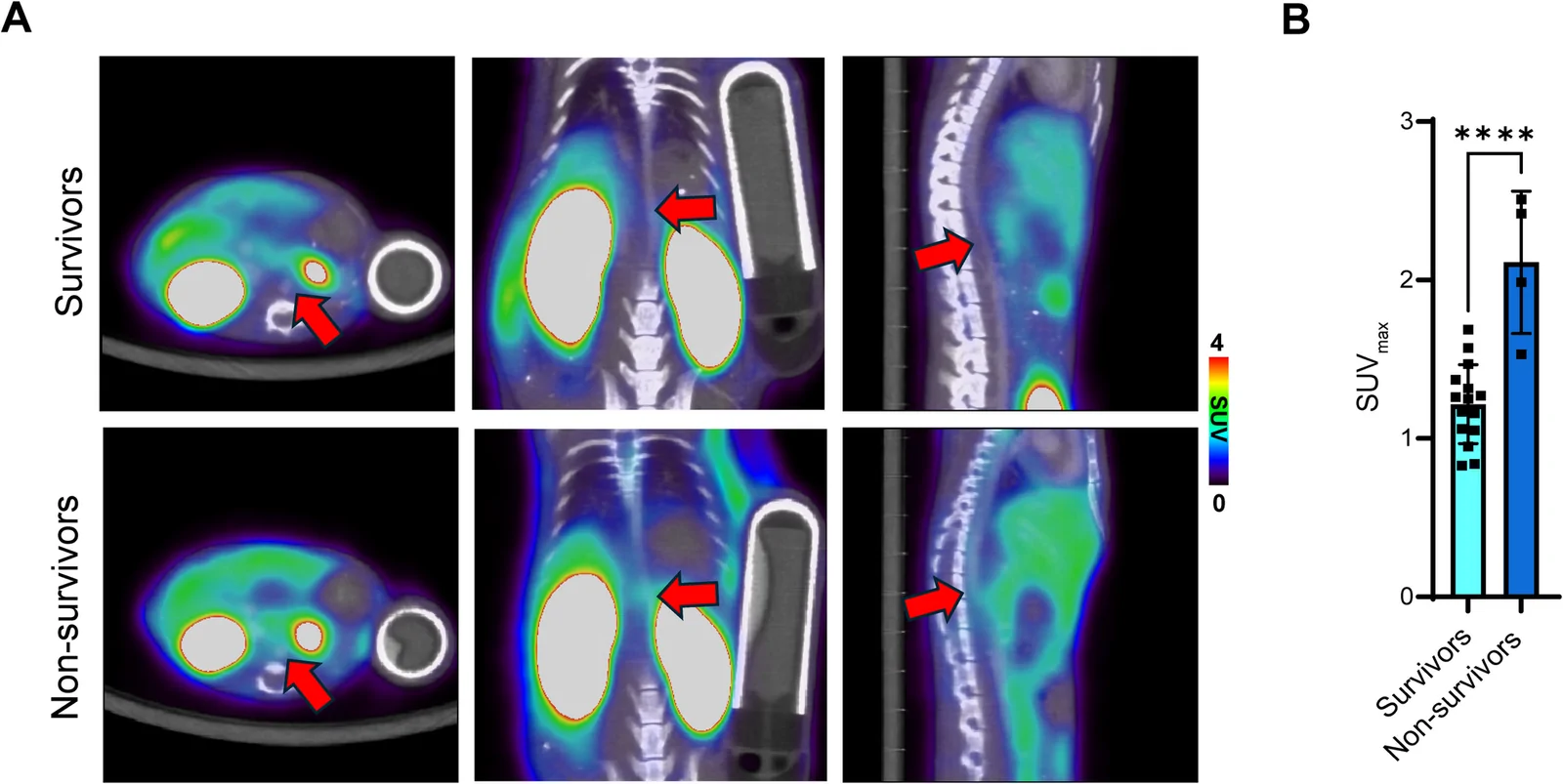
^64^Cu-RYM2 PET/CT and survival in angiotensin II-infused *Apoe*^*−/−*^ mice. (**A**) Illustrative examples of fused ^64^Cu-RYM2 PET/contrast-enhanced CT images (axial, coronal, and sagittal) acquired 7 days after starting angiotensin II infusion in young (8–10 weeks) mice that survived to day 28 (survivors) and from mice that died before day 28 (non-survivors). Red arrows indicate the suprarenal abdominal aorta. (**B**) Quantification of the suprarenal abdominal aorta ^64^Cu-RYM2 signal on PET/CT images acquired on day 7 in young and old non-survivors and survivors. ****: *P* < 0.0001. SUV: Standardized uptake value

In vivo PET/CT imaging with ^64^Cu-RYM2 (a tracer targeting activated MMPs [11]) on day 7 of Ang II infusion showed significantly higher aortic tracer uptake in old mice than in young animals [descending thoracic aorta (DTA): SUVmax 0.96 ± 0.20 in young mice vs. 1.36 ± 0.39 in old mice, *P* < 0.01; suprarenal abdominal aorta (SRA): SUVmax 1.17 ± 0.23 in young mice vs. 1.80 ± 0.52 in old mice, *P* < 0.01; Fig. 2 and Supplemental Fig. 2]. The blood radioactivity measured in the left ventricle was also significantly different between the two groups (SUVmean 0.60 ± 0.28 in old mice vs. 0.40 ± 0.11 in young mice, *P* < 0.05, Supplemental Fig. 3). Notably, suprarenal abdominal aortic tracer uptake was significantly higher in animals that did not survive 28 days than in those that did (SUVmax 1.21 ± 0.25 in surviving animals vs. 2.11 ± 0.45 in non-surviving animals, *P* < 0.0001, Fig. 3 and Supplemental Fig. 4). Furthermore, there was a significant association between SUVmax values as a dichotomous variable (using a cut-off of 1.5) and survival, with 13 out of 13 (100%) mice with an SUVmax < 1.5 and 2 out of 6 (33.3%) mice with an SUVmax ≥ 1.5 surviving to 28 days (Fisher’s exact test *P* = 0.0039).

**Fig. 4:**
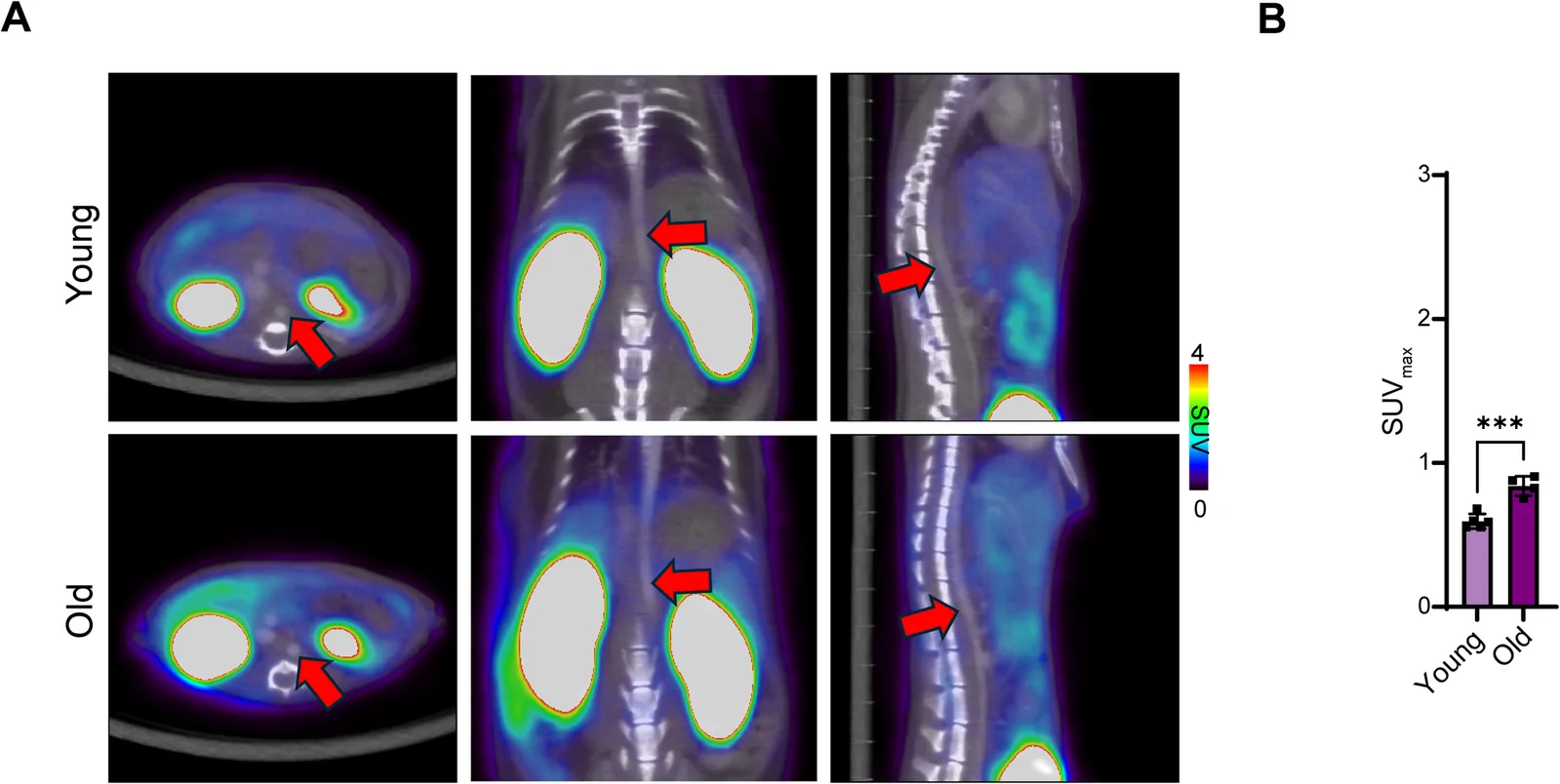
Age and ^64^Cu-RYM2 PET/CT in control *Apoe*^*−/−*^ mice. **(A)** Illustrative examples of fused ^64^Cu-RYM2 PET/contrast-enhanced CT images (axial, coronal, and sagittal) acquired in control young (8–10 weeks) and old (> 51 weeks) *Apoe*^*−/−*^ mice. Red arrows indicate the suprarenal abdominal aorta. **(B)** Quantification of the ^64^Cu-RYM2 signal in the suprarenal abdominal aorta in young and old mice. ***: *P* < 0.001. SUV: Standardized uptake value

To determine whether the higher aortic MMP activation in Ang II-infused old mice compared with young animals persists without Ang II infusion, we compared the ^64^Cu-RYM2 signal on PET/CT images of a previously reported group of young *Apoe*^*−/−*^ mice with the aortic signal of old *Apoe*^*−/−*^ mice undergoing ^64^Cu-RYM2 PET/CT imaging. This showed significantly higher suprarenal abdominal aortic tracer uptake in old mice than in young animals (SUVmax 0.60 ± 0.05 in young mice, vs. 0.84 ± 0.07 in old mice, *P* < 0.001, Fig. 4). In both young and old animals, the suprarenal abdominal aortic MMP signal on ^64^Cu-RYM2 PET/CT images was significantly higher after 7 days of Ang II infusion than in control mice (*P* < 0.0001 for young and *P* < 0.01 for old animals, Supplemental Fig. 5).

### Inflammation, MMP activity, and aortic dissection

**Fig. 5:**
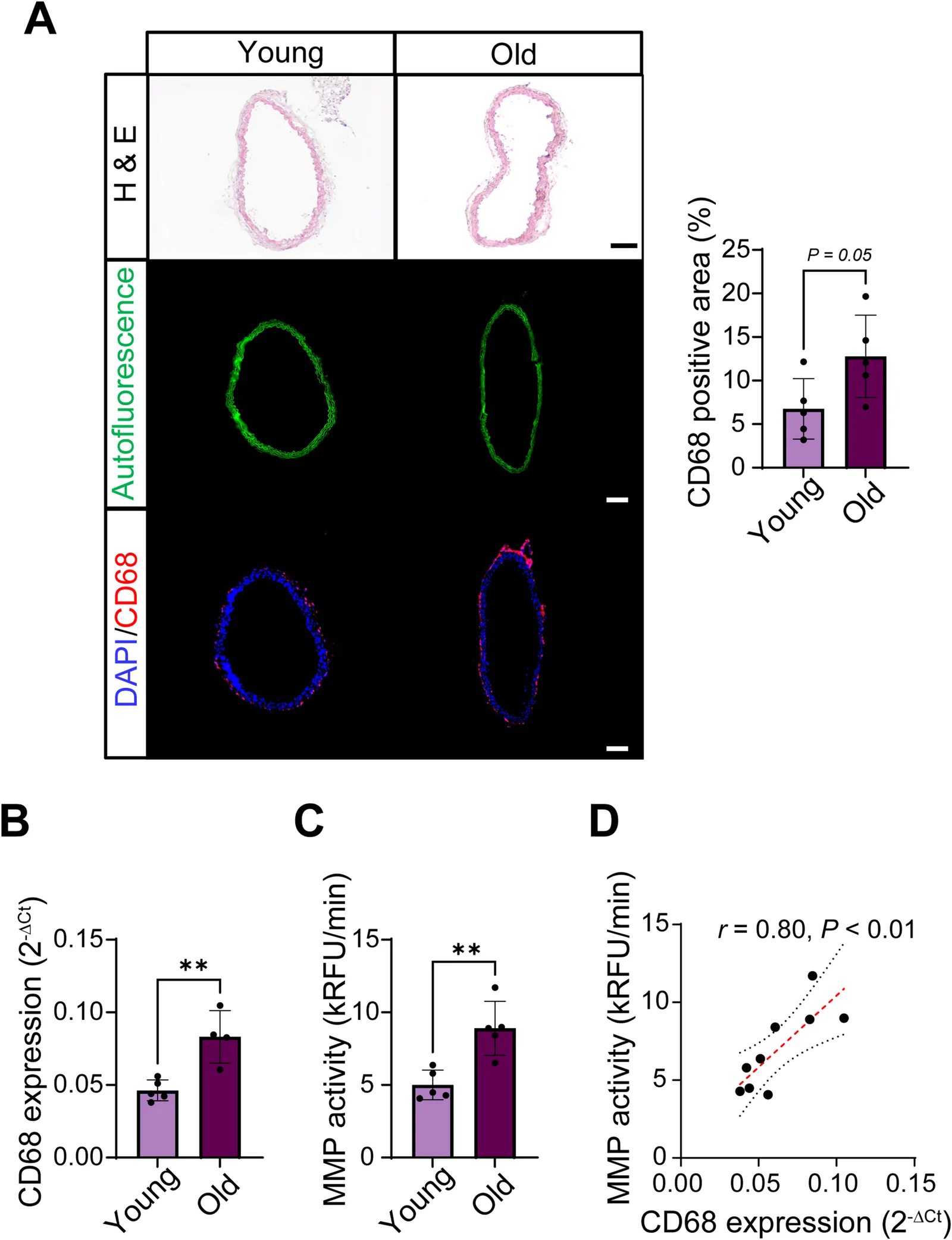
Aortic inflammation and MMP activity in angiotensin II-infused *Apoe*^*−/−*^ mice. Illustrative examples of suprarenal abdominal aorta hematoxylin and eosin (H & E) staining, autofluorescence, and CD68 expression and its quantification by immunostaining (**A**), aortic *Cd68* expression by quantitative RT-PCR (**B**), and aortic MMP activity (**C**) in young and old *Apoe*^*−/−*^ mice after 3 days of angiotensin II infusion. **D**. Correlation between aortic *Cd68* gene expression and MMP activity. Scale bar: 100 μm, RFU: Relative fluorescence units

As reported previously, Ang II infusion promoted aortic macrophage infiltration (Supplemental Fig. 6). Quantification of CD68 expression showed higher macrophage infiltration in old mice than in young animals after 3 days of Ang II infusion (Fig. 5A, B). In line with ^64^Cu-RYM2 imaging data, aortic MMP activity assessed by tissue zymography at this early time point, when dissection is rarely observed, was significantly higher in old mice than in young animals (Fig. 5C) and significantly correlated with *Cd68* expression (*r* = 0.80, *P* < 0.01, Fig. 5D). Consistent with blood ^64^Cu-RYM2 radioactivity levels, evaluation of MMP activity in blood cells showed significantly higher MMP activity in old mice than in young animals (Supplemental Fig. 7).

The development of an aortic aneurysm following Ang II infusion is preceded by focal medial dissections that present as intramural hematoma, and ultimately evolve into aneurysmal aortic remodeling [17]. To investigate the timing of increased aortic MMP activity after Ang II infusion in relation to medial dissection and aortic remodeling, we evaluated the aorta and its MMP activity in fully mature, intermediate-age (12- to 17-week-old) *Apoe*^*−/−*^ mice before and after Ang II infusion. Notably, while there was seldom any evidence of aortic dissection along the aorta before day 3, a subset of aortas collected on day 3 and day 4, showed early dissection as evidenced by intramural hematoma in the SRA (Supplemental Fig. 8). Tissue zymography of intact aortas showed a significant increase in aortic MMP activity on day 3 after Ang II infusion compared to control animals (Supplemental Fig. 9). Together, these data suggest that increased MMP activity in the aorta precedes aortic dissection, an early stage in aneurysm development and rupture in this model.

### Heterogeneity of MMP activity along the aorta

**Fig. 6:**
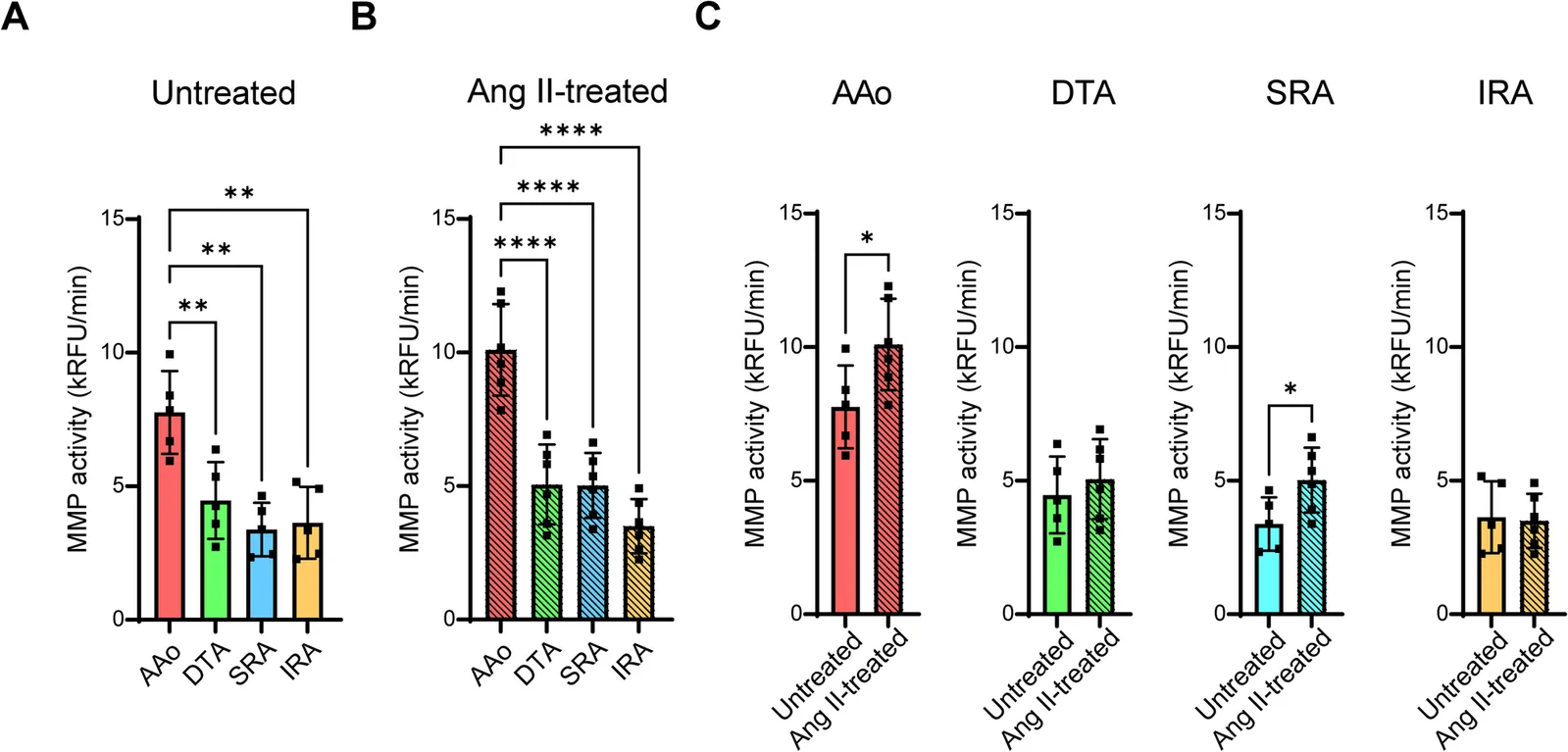
MMP activity in different segments of the aorta of *Apoe*^*−/−*^ mice (12–17 weeks). **A**,** B.** MMP activity in different aorta segments from control *Apoe*^*−/−*^ mice (**A**), and *Apoe*^*−/−*^ mice infused with angiotensin II for 3 days (**B**). **C.** Comparison of MMP activity between control and angiotensin II-infused mice within each segment of the aorta. *:*P* < 0.05, **: *P* < 0.01, ****: *P* < 0.0001. RFU: Relative fluorescence units, AAo: Ascending aorta, DTA: Descending thoracic aorta, SRA: suprarenal abdominal aorta, IRA: Infrarenal abdominal aorta

Given the SRA location of aortic dissection, we evaluated the pattern of MMP activity along the aorta before and early after Ang II infusion in 12- to 17-week-old *Apoe*^*−/−*^ mice. To generate a detailed map of MMP activity along the aorta, we compared two molecular imaging approaches, fluorescence imaging with MMPSense 645 (a panMMP substrate that fluoresces upon cleavage by MMPs) and autoradiography with ^99m^Tc-RYM1 (a ^99m^Tc-labeled ^64^Cu-RYM2 homolog tracer that, like RYM2, binds the common MMP activation epitope), with tissue zymography. Although all three techniques showed higher MMP activity in the AAo, only the difference detected by tissue zymography reached statistical significance (MMP activity in AAo compared with DTA (*P* < 0.01), SRA (*P* < 0.01), or infrarenal aorta (IRA, *P* < 0.01; Supplemental Figs. 10 and Fig. 6A). MMP activity was also higher in the AAo than in other segments of the aorta in animals infused with Ang II for 3 days [MMP activity in the AAo compared with DTA (*P* < 0.0001), SRA (*P* < 0.0001), or IRA (*P* < 0.0001); Fig. 6B]. However, the Ang II-induced increase in MMP activity was not uniform along the aorta; the AAo and SRA, but not the DTA and IRA, showed significant increases in MMP activity on day 3 after Ang II infusion (*P* < 0.05 for AAo and SRA; Fig. 6C). To investigate the effect of aging on heterogeneity in MMP activity along the aorta, similar studies were performed in 8-10-week-old and > 51-week-old *Apoe*^*−/−*^ mice. Higher AAo MMP activity was also observed in 8–10-week-old animals. Additionally, aging was associated with a significant increase in MMP activity in AAo (*P* < 0.05, Supplemental Fig. 11).

### Aging and the heterogeneity of MMP activity along the aorta in WT mice

**Fig. 7:**
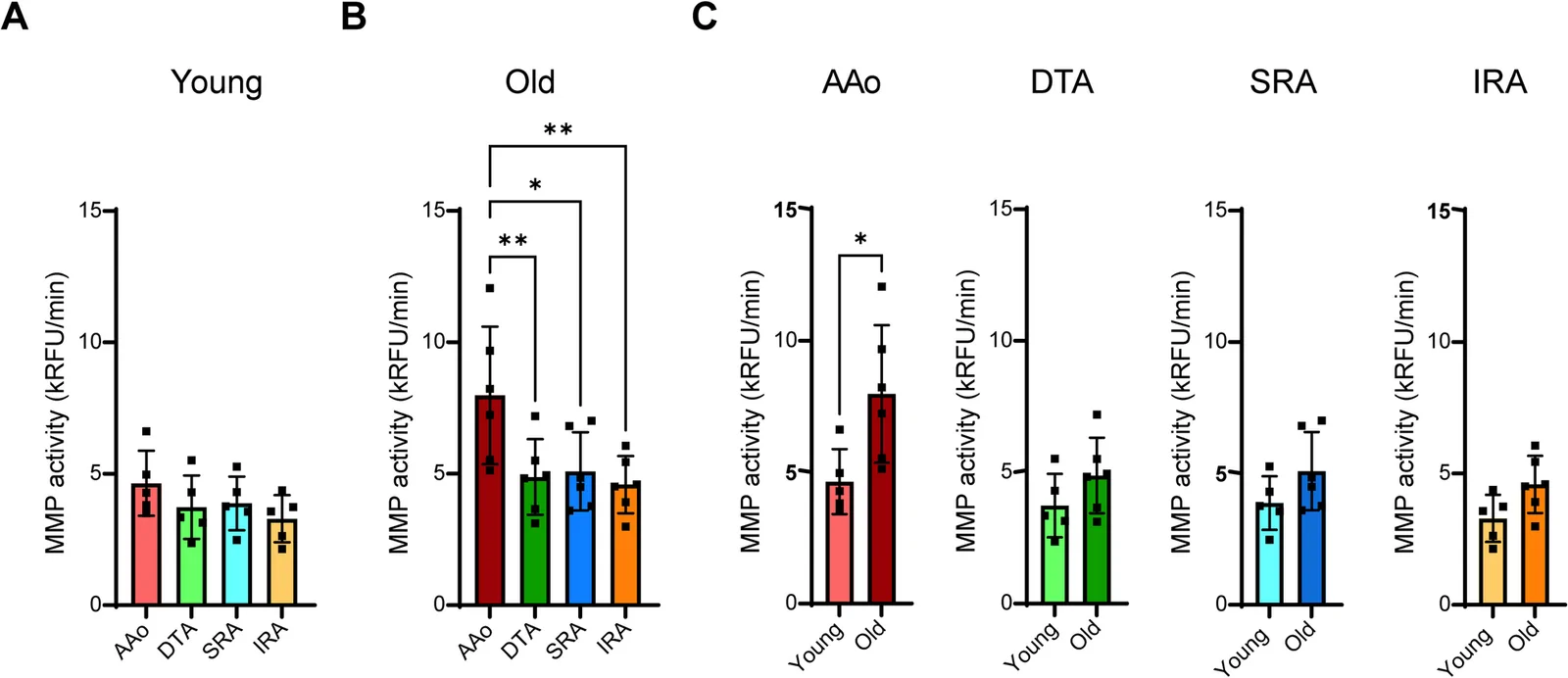
MMP activity in different segments of the aorta of wild-type mice. **A**,** B.** MMP activity in different aorta segments from young (8–10 weeks, **A**), and old (> 51 weeks) mice (**B**). **C.** Comparison of MMP activity between young and old mice within each segment of the aorta. *: *P* < 0.05, **: *P* < 0.01. RFU: Relative fluorescence units, AAo: Ascending aorta, DTA: Descending thoracic aorta, SRA: suprarenal abdominal aorta, IRA: Infrarenal abdominal aorta

To investigate whether a similar heterogeneous pattern of MMP activity exists in WT mice, we assessed MMP activity by tissue zymography in 8-10-week-old (young) and > 51-week-old (old) WT mice. While there was no difference in MMP activity among segments of the aorta in young mice, older WT mice showed heterogeneous MMP activity along the aorta, with significantly higher MMP activity in the AAo than in the other three segments (*P* < 0.01, 0.05, and 0.01, respectively, for the DTA, SRA, and IRA; Fig. 7A and B). Comparing MMP activity across aortic segments between these two animal groups showed a trend toward increased MMP activity with aging, which was statistically significant in the AAo (*P* < 0.05, Fig. 7C). A comparison of MMP activity between WT and *Apoe*^*−/−*^ mice showed higher activity in the AAo of *Apoe*^*−/−*^ mice in both young and old animals. In young mice, MMP activity was also significantly higher in the SRA of *Apoe*^*−/−*^ mice than in WT animals (Supplemental Fig. 12).

## Discussion

This study offers new insight into the role of MMPs in aortic aneurysm. Aging is associated with decreased survival in mice infused with Ang II to induce aortic aneurysm, as well as with increased aortic MMP activation detected by molecular imaging before and after Ang II infusion. Animal survival is associated with lower MMP signals in early in vivo PET/CT images after Ang II infusion. Additionally, aging is associated with increased MMP activity, which correlates with aortic inflammation, and its heterogeneity along the aorta.

Advanced age is a risk factor for the sporadic form of TAA and AAA [2, 19]. Age may also influence aneurysm evolution. Although human data on TAA are inconsistent in this regard [2], the incidence of AAA rupture increases with age [20]. Only a small number of preclinical studies have formally examined the impact of age on AAA formation and outcomes in controlled settings [21]. A study comparing AAA development in Ang II-infused 10-week-old and 24-week-old *Apoe*^*−/−*^ mice reported a higher AAA-to-normal aortic diameter ratio in the 24-week-old group [22]. Another study reported a higher incidence of AAA and lower survival in Ang II-infused C57BL/6 mice aged 18 to 20 months compared with those aged 2 to 3 months [23]. Our study confirms and extends these findings, demonstrating a significant reduction in survival in old versus young animals, associated with enhanced MMP activation detected by molecular imaging.

MMPs can be produced by different cells, e.g., macrophages, within the vascular wall, and several studies have reported higher MMP expression in aortic aneurysms than in the normal aorta [10, 11]. However, expression levels do not necessarily correlate with proteolytic function (MMP activity), which more directly reflects the matrix-degrading environment that drives the progression of aortic aneurysms [9]. We leveraged MMP-targeted molecular imaging to investigate how animal age affects aortic MMP activation in control and Ang II-infused mice. ^64^Cu-RYM2 (a tracer that binds to the common MMP activation epitope [11]) PET/CT imaging showed higher aortic tracer uptake in surviving old mice than in young animals after 1 week of Ang II infusion. A similar difference in MMP tracer uptake between old and young mice was observed in control animals, and Ang II infusion increased tracer uptake in both groups. Notably, the SRA tracer uptake was significantly higher in animals that did not survive to 28 days than in those that did, and there was a significant association between SUVmax values as a dichotomous variable and survival, suggesting that aortic MMP activation may contribute to aneurysm development and rupture, and can serve as a survival marker.

A few studies have reported differences in MMP expression across regions of the aorta and in surgical specimens obtained after aortic dissection or aneurysm rupture [10, 24]. However, no systematic evaluation of MMP activity along the aorta has been reported. The aorta is a complex organ with significant differences in the structural and biomechanical properties of its regions, which may respond differently to aging and injury [24–26]. The decrease in aortic size along its course is associated with key differences in the number of lamellar units separated by elastic laminae between the thoracic and abdominal aorta [24], and changes in aortic stiffness, which gradually increases along the aorta [27, 28]. The transition from disturbed to laminar flow, the change in blood flow direction in the aortic arch, and the presence of side branches along the descending aorta also contribute to the biomechanical heterogeneity of the aorta. Smooth muscle cells (SMC) contribute to the integrity of the aortic wall, and their loss is a key feature in the pathogenesis of AAA. There are significant differences in the embryologic origins of SMC along the aorta: those in the proximal thoracic aorta (including the aortic arch) derive from the neural crest and the secondary heart field. In contrast, SMC in the remainder of the aorta are mesodermal derivatives [29, 30]. Analysis of MMP activity along the mouse aorta reveals a similar heterogeneity that becomes more pronounced with aging and Ang II infusion.

*Apoe*^*−/−*^ mice are prone to atherosclerosis, and we cannot exclude the possibility that the higher AAo MMP activity observed in older *Apoe*^*−/−*^ mice is partly related to atherosclerosis in this atheroprone segment of the aorta. However, the higher AAo MMP activity in old wild-type mice links aging to increased AAo MMP activity, independent of atherosclerosis. The higher MMP activity in *Apoe*^*−/−*^ mice compared with wild-type animals, which may be influenced by atherosclerosis, may also contribute to their increased propensity for aneurysm development [21]. Notably, the increase in MMP activity in Ang II-infused *Apoe*^*−/−*^ mice is restricted to the AAo and SRA segments, which are more prone to aneurysm formation, dissection, and rupture [21, 31, 32]. The higher predilection of the SRA relative to the AAo for developing dissecting aneurysms may be linked to an early mural tear near the ostium of small suprarenal side branches and local destruction of the medial architecture, as detected by differential phase-contrast X-ray tomographic microscopy and histology [33]. Moreover, the earliest evidence of aortic aneurysm and dissection, i.e., intramural hematoma, is detected in the SRA on days 3–4 of Ang II infusion, suggesting that MMP activation in this segment may set the stage for subsequent events.

### Limitations

We cannot rule out that differences in tracer clearance have contributed to the observed difference in the MMP signal between young and old animals on PET/CT images. However, higher MMP activity in old animals was also observed using an independent technique, tissue zymography. MMP activity depends on MMP expression, MMP activation, and the presence of MMP inhibitors [9]. A detailed assessment of the expression and activation of various members of the MMP family and their tissue inhibitors will be the subject of future studies. In the absence of radiotracers and other agents highly selective for specific MMPs, we could only evaluate the overall MMP activation in our longitudinal studies of MMP activation and aneurysm outcomes.

There are key differences between the pathogenesis of TAA and AAA in humans, with dissection being more common in TAA, while rupture occurs more frequently in AAA. Dissection and medial accumulation of macrophages precede the development of aneurysm in the Ang II infusion model [17, 21]. This sequence of events is somewhat different from a classic human AAA, in which early dissection is infrequent. Therefore, this model does not completely mimic human disease. The same observation holds for other preclinical models of AAA, such as elastase and CaCl_2_-induced aneurysms, in which the sequence of events differs from that in human disease [34]. Despite its limitations, the Ang II infusion model is extensively employed as an experimental model of AA [21]. Ultimately, any findings in preclinical models of AA need validation in humans.

## Conclusion

Aging is associated with increased MMP activity and heterogeneity along the aorta, which may underlie the lower aneurysm survival in older animals. Highlighting the role of MMPs in aneurysm pathobiology, molecular imaging of MMP activation provides insights regarding aneurysm survival prospects in this exploratory cohort. Taken together, these data suggest that combining MMP inhibitors and tracers, whether global or targeting specific MMP family members, may help prevent and track aneurysm growth, dissection, and rupture.

## Supplementary Information

Below is the link to the electronic supplementary material.

Supplementary Material 1

## Data Availability

The datasets generated during and/or analyzed during the current study are available from the corresponding author on reasonable request.

## Abbreviations

AA: Aortic aneurysm
AAA: Abdominal aortic aneurysm
AAo: Ascending aorta
Ang II: Angiotensin II
ApoE: Apolipoprotein E
DTA: Descending thoracic aorta
IRA: Infrarenal abdominal aorta
MMP: Matrix metalloproteinase
PET: Positron emission tomography
SPECT: Single photon emission computed tomography
SRA: Suprarenal abdominal aorta
TAA: Thoracic aortic aneurysm
VOIs: Volumes of interest
WT: Wild type
