# Supplementary material for: Matrix metalloproteinase imaging and survival prospects with aging in murine aortic aneurysm
Source: EJNMMI Res. 2026 Sep 22;16:146. doi: 10.1186/s13550-026-01521-w (PMC13675461; doi:10.1186/s13550-026-01521-w)
Supplement: Supplementary file 1 [file 13550_2026_1521_MOESM1_ESM.pdf]

Aging, matrix metalloproteinase imaging, and survival prospects with aging in aortic aneurysm

Mean Ghim, PhD[1], Onur Varli, MD[1], Azmi A. Ahmad, PhD[1,2], Afarin Neishabouri, MD[1,2], Gunjan Kukreja, PhD[1,2], Zhengxing Zhang, PhD[1], Saalar Zarnegar[1], Nicholas Gangemi[1], Keshvad Hedayatyanfard, DVM, PhD[1], Jakub Toczek, PhD[1,2], Lingyun Wang, PhD[1,2], Jiasheng Zhang, MD[1], Chi Liu, PhD[3], Yongjian Liu, PhD[4], Robert J. Gropler, MD[4], Mehran M. Sadeghi, MD[1,2]

[1] Yale Cardiovascular Research Center, Section of Cardiovascular Medicine, Department of Internal Medicine, Yale School of Medicine, New Haven, CT, USA

[2] Veterans Affairs Connecticut Healthcare System, West Haven, CT, USA

[3] Department of Radiology and Biomedical Imaging, Yale University, New Haven, CT, USA

[4] Department of Radiology, Washington University, St. Louis, MO, USA

Supplemental Data

Table of Contents

Supplemental Methods	3
Supplemental Figure 1	5
Supplemental Figure 2	6
Supplemental Figure 3	7
Supplemental Figure 4	8
Supplemental Figure 5	9
Supplemental Figure 6	10
Supplemental Figure 7	11
Supplemental Figure 8	12
Supplemental Figure 9	13
Supplemental Figure 10	14
Supplemental Figure 11	15
Supplemental Figure 12	16

Supplemental Methods

Morphometry

5-μm-thick serial cross-sections of the abdominal aorta were used for morphometric analysis. The circumference of the external elastic lamina in each section was measured, and the maximum diameter was calculated assuming a circular geometry. A maximum diameter of > 1.5 mm was considered indicative of an aortic aneurysm.

Hematoxylin and Eosin (H&E), and CD68 staining

OCT-embedded aortic segments were sectioned at 5 μm and mounted on glass slides. OCT was removed by washing sections in PBS, and the tissue was fixed in 10% neutral buffered formalin for 15 minutes. Slides were stained with hematoxylin (Abcam) for 15 seconds, rinsed in two changes of distilled water, and incubated with a bluing reagent (Abcam) for 10 seconds. They were then briefly dipped in absolute alcohol, counterstained with modified alcoholic eosin Y (Abcam) for 3 minutes, rinsed in absolute alcohol, dehydrated through three changes of absolute alcohol, and mounted. Brightfield images were acquired with a light microscope (Bx63; Olympus). For CD68 immunostaining, SRA cross-sections were fixed in 4% paraformaldehyde for 15 minutes, blocked with 5% bovine serum albumin in PBS for 1 h, and incubated with rat anti-mouse CD68 primary antibody (Abcam) for 1 h at room temperature, followed by Alexa Fluor 595-conjugated anti-rat antibody (Thermo Fisher Scientific) for 1 h at room temperature. Nuclei were counterstained with 4′,6-diamidino-2-phenylindole (DAPI) for 15 min. Sections were imaged using a Leica DMi8 microscope, and the CD68-positive area was quantified as a percentage of the total aortic cross-sectional area from two non-adjacent sections per animal, and the values were averaged. In each section, the outer boundary of the tissue defined the total aortic cross-sectional area, and CD68 fluorescence was thresholded using a uniform threshold applied across all images to distinguish the CD68 signal from background.

Reverse transcription polymerase chain reaction (RT-PCR)

Total RNA was isolated from aortic tissues, and cDNA was synthesized using the RNeasy Plus Mini Kit and the QuantiTect Reverse Transcription Kit (Qiagen) according to the manufacturer's instructions. Real-time quantitative PCR was performed on a CFX96 Real-Time PCR System (Bio-Rad). Primer sequences were as follows: *Cd68* forward, 5′-CCTCTTGCTGCCTCTCATCATTGG-3′, and reverse, 5′-GGCTGGTAGGTTGATTGTCGTCTG-3′; *Gapdh* forward, 5′-

GTATGACTCCACTCACGGCAAA-3′, and reverse, 5′-GGTCTCGCTCCTGGAAGATG-3′. Gene expression was reported as $2^{-\Delta Ct}$ relative to *Gapdh*.

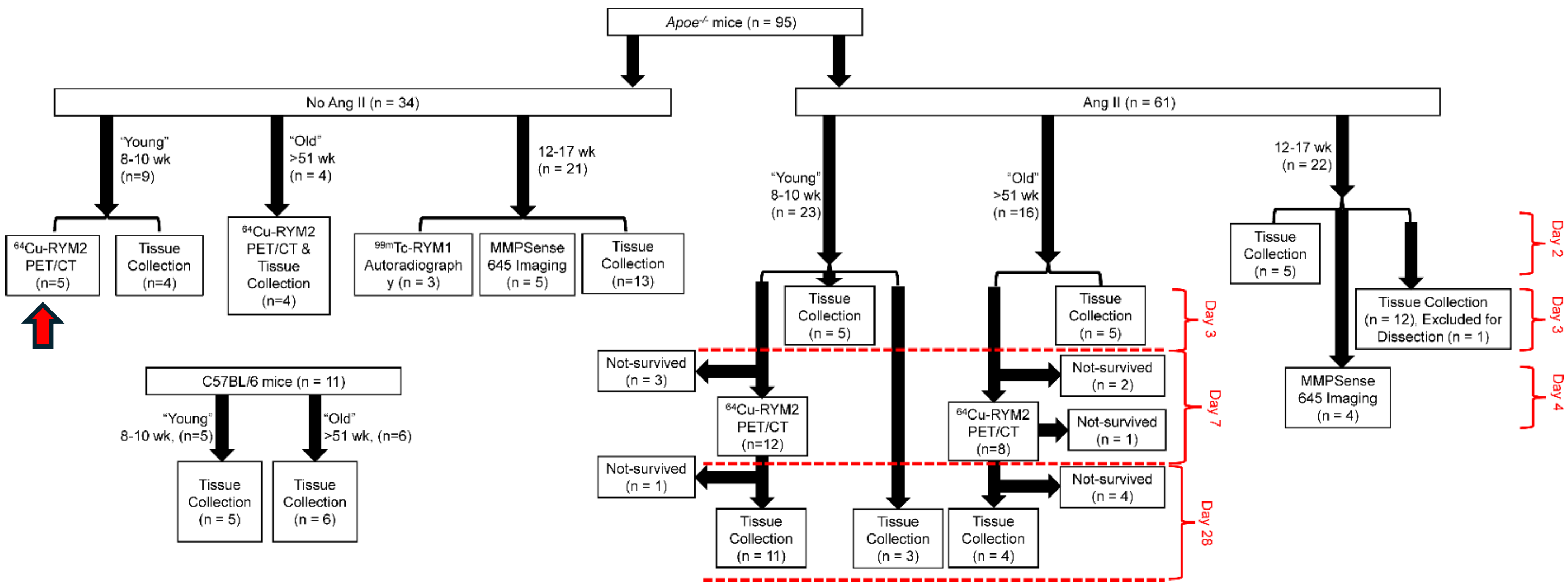

Supplemental Figure 1. Flow chart of animals used in the study and their endpoints. Animal survival was tracked in young (8-10 weeks) and old (>51 weeks) *Apoe*$^{-/-}$ mice infused with angiotensin II (Ang II) for up to 28 days. The animals underwent in vivo ^{64}Cu-RYM2 PET/CT imaging after 7 days to evaluate aortic MMP activation in relation to survival to 28 days. The temporal and spatial patterns of MMP activity and its relation to aortic dissection, an early step in aneurysm development in this model, was investigated by tissue zymography, and ex vivo molecular imaging in an intermediate age, fully adult (12-17 weeks) group of *Apoe*$^{-/-}$ mice infused with Ang II for up to 4 days and the effect of aging on these findings was corroborated in aortas collected from young and old *Apoe*$^{-/-}$ mice after 3 days of Ang II infusion. Age-matched non-Ang II-infused *Apoe*$^{-/-}$ mice and young (8-10 weeks) and old (>51 weeks) C57BL/6 mice were used as controls in different experiments. A previously acquired PET/CT dataset of young *Apoe*$^{-/-}$ mice (n = 5, arrow) [reference 11] was reanalyzed for use in the current study.

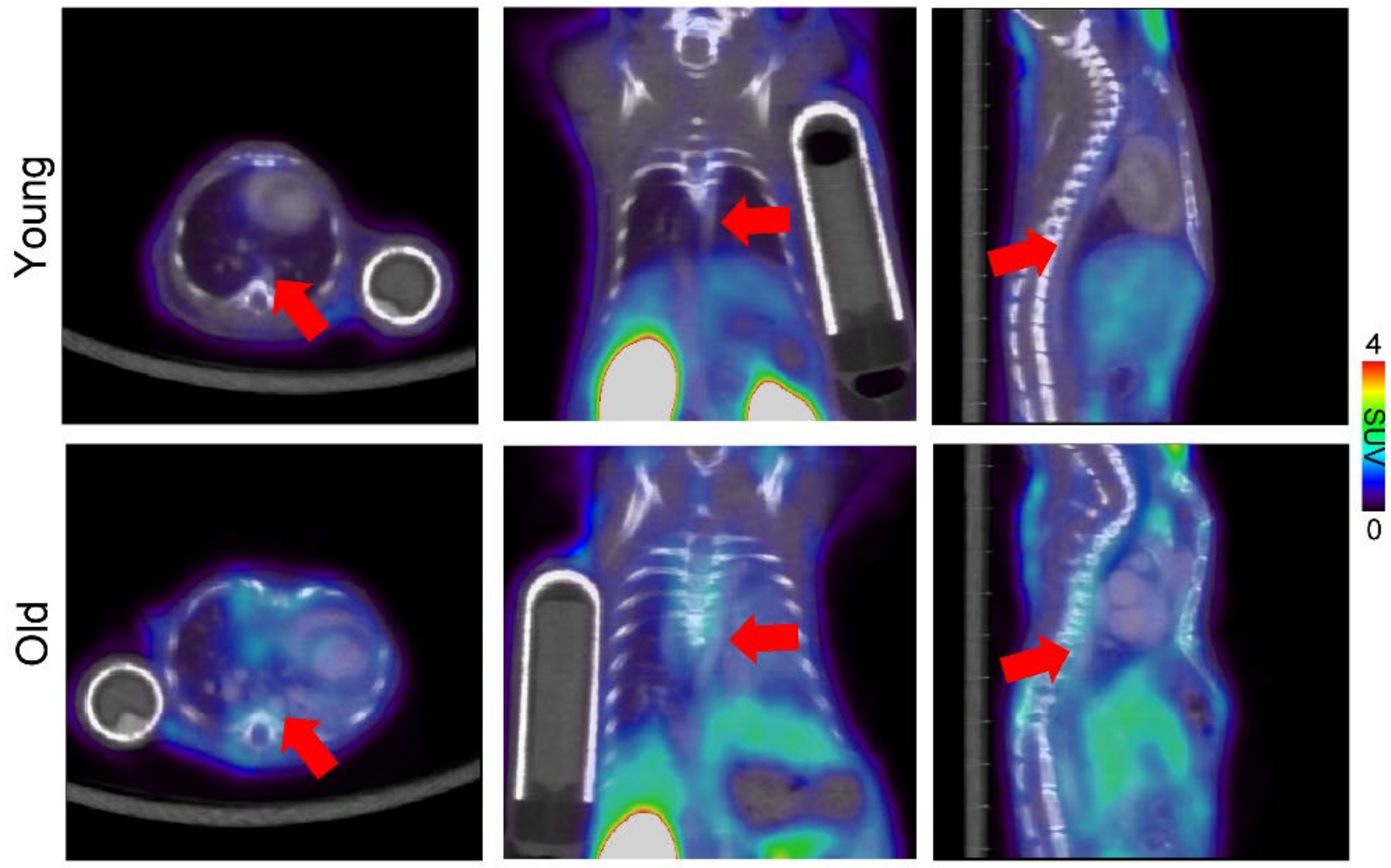

Supplemental Figure 2. Illustrative examples of fused ^{64}Cu-RYM2 PET/contrast-enhanced CT images (axial, coronal, and sagittal) acquired 7 days after starting angiotensin II infusion in young (8–10 weeks) and old (>51 weeks) *Apoe*$^{-/-}$ mice. Red arrows indicate the descending thoracic aorta.

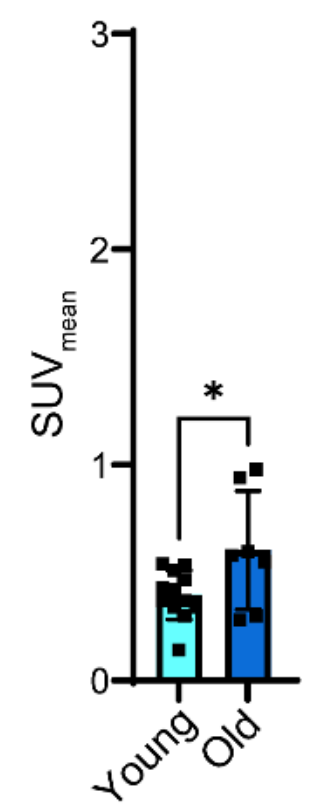

Supplemental Figure 3. Quantification of the ^{64}Cu-RYM2 blood activity measured in the left ventricle on PET/CT images acquired in 7 days angiotensin II-infused *Apoe*$^{-/-}$ mice. *: $P < 0.05$. SUV: Standardized uptake value.

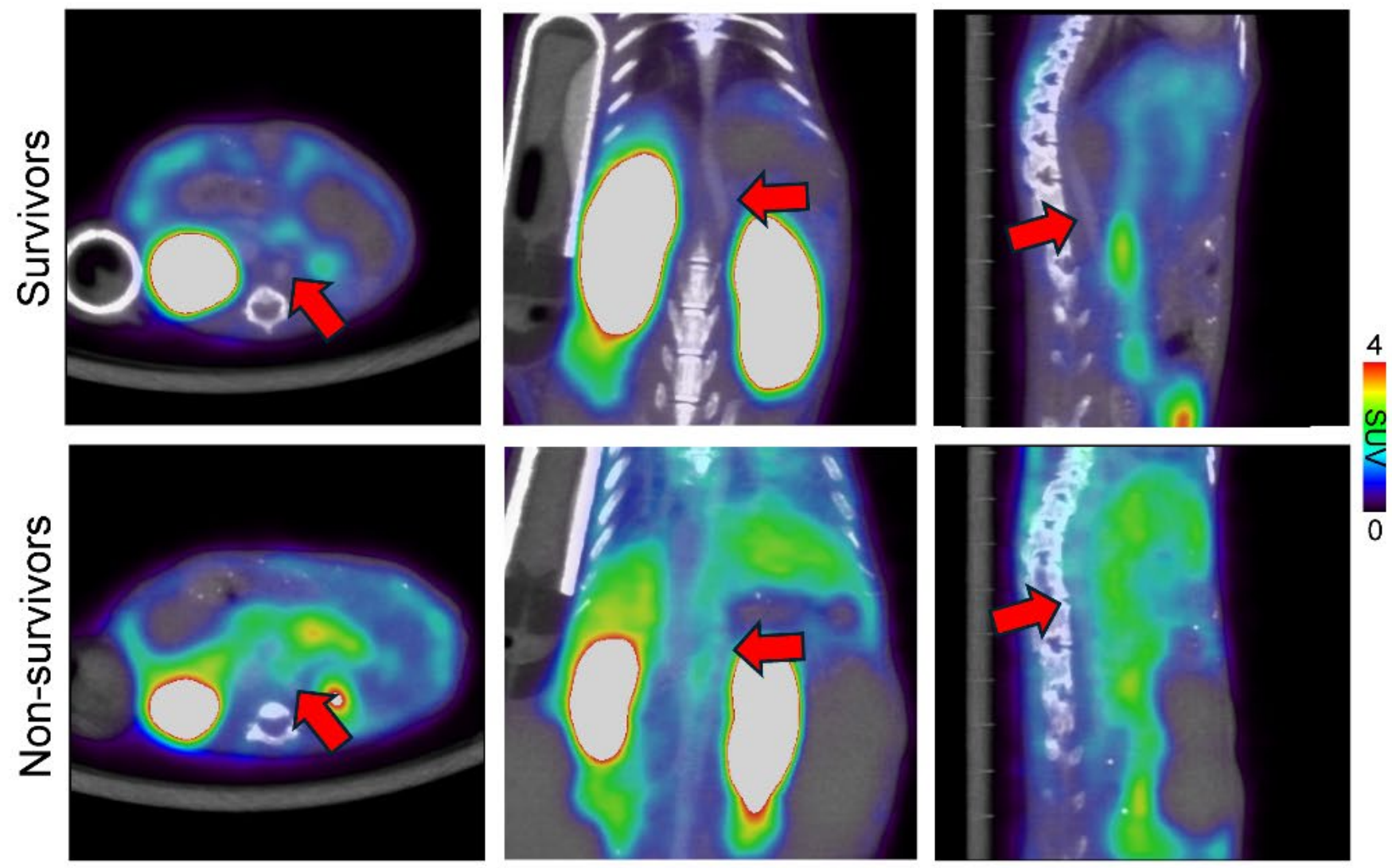

Supplemental Figure 4. Illustrative examples of fused ^{64}Cu-RYM2 PET/contrast-enhanced CT images (axial, coronal, and sagittal) acquired 7 days after starting angiotensin II infusion in old (>51 weeks) mice that survived to day 28 (survivors) and from mice that died before day 28 (non-survivors). Red arrows indicate the suprarenal abdominal aorta.

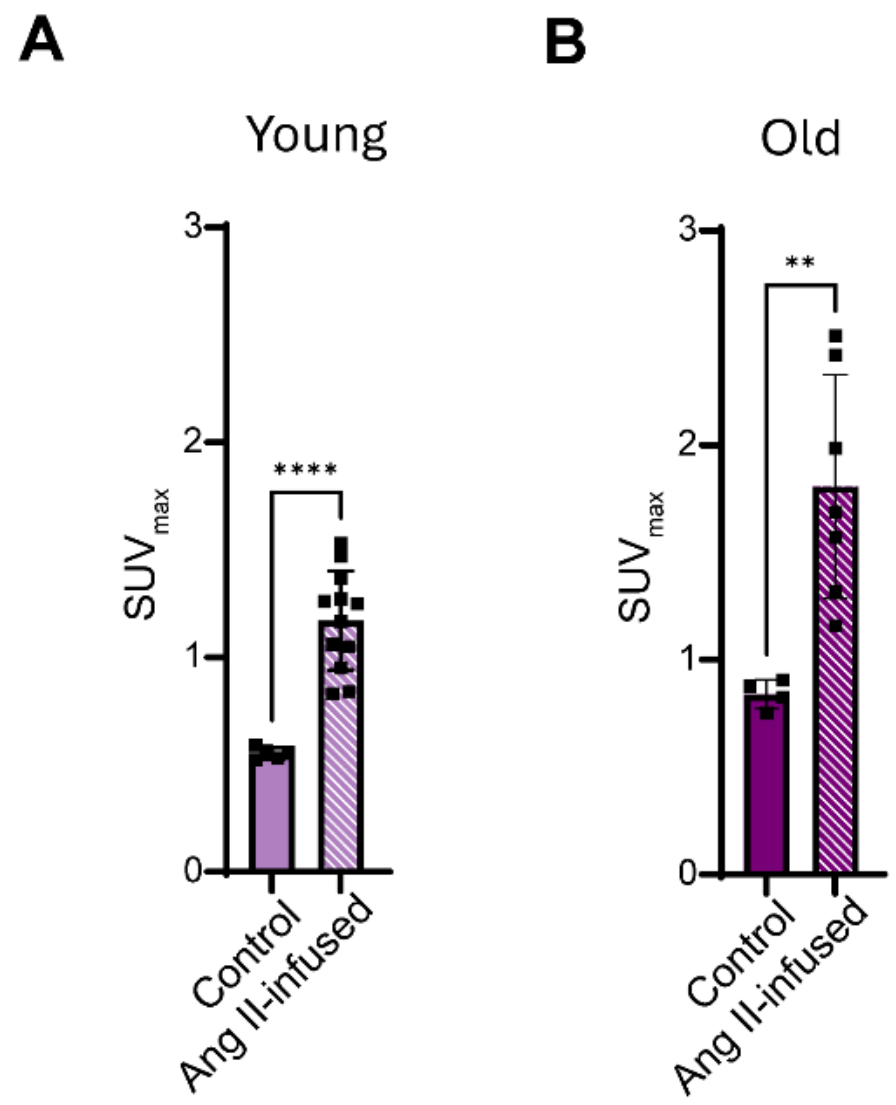

Supplemental Figure 5. Quantification of the suprarenal abdominal aorta ^{64}Cu-RYM2 signal on PET/CT images acquired in control, and 7 days angiotensin II-infused young (8-10 weeks, **A**) and old (>51 weeks, **B**) *Apoe*$^{-/-}$ mice. **: *P* < 0.01, ****: *P* < 0.0001. SUV: Standardized uptake value.

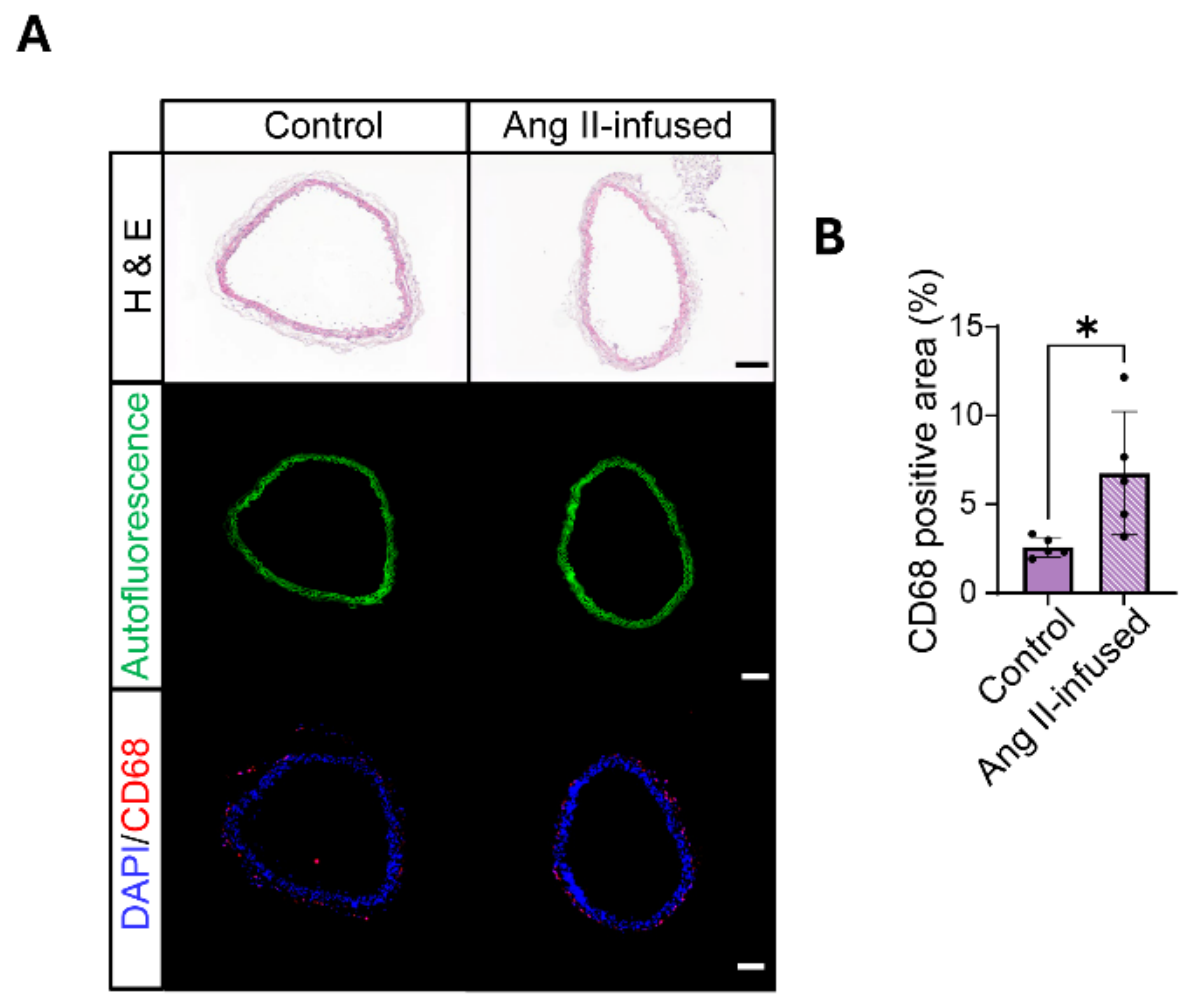

Supplemental Figure 6. Illustrative examples of suprarenal abdominal aorta hematoxylin and eosin (H & E) staining, autofluorescence, and CD68 immunostaining (**A**) and quantification of CD68 staining (**B**) in young control and angiotensin II-infused *Apoe*$^{-/-}$ mice after 3 days. Scale bar: 100 μm.

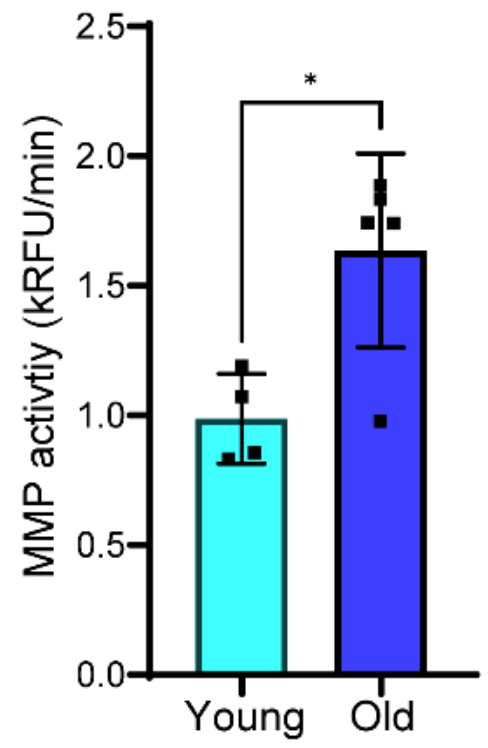

Supplemental Figure 7. Quantification of MMP activity in blood cells of young (8-10 weeks) and old (8-9 months) *Apoe*$^{-/-}$ mice. *: $P < 0.05$. RFU: Relative fluorescence units.

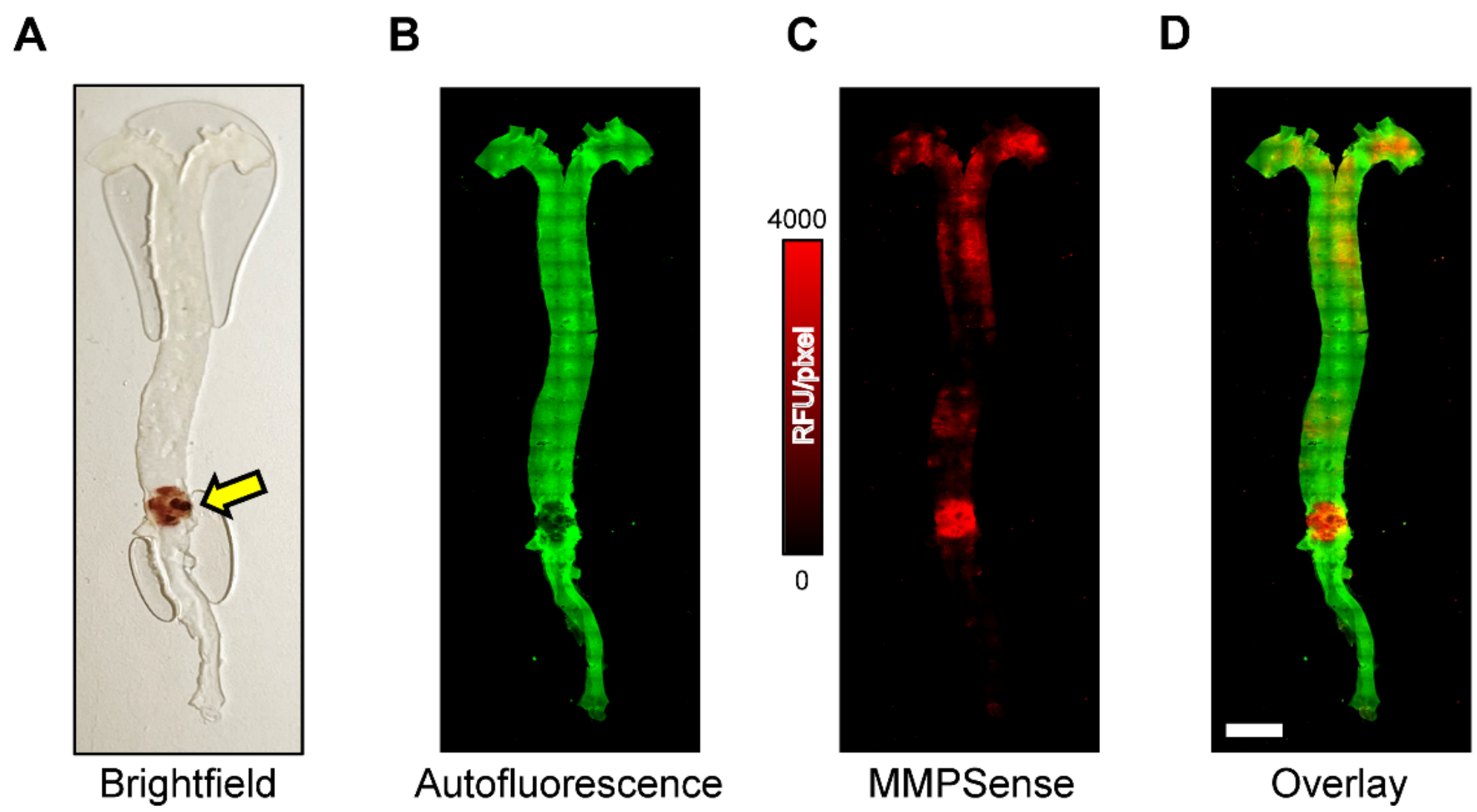

Supplemental Figure 8. Illustrative brightfield (**A**), autofluorescence (**B**), MMPSense 645 fluorescence (**C**), and fused MMPSense 645 fluorescence/autofluorescence (**D**) whole-mount images of the aorta of an angiotensin II-infused (for 4 days) *Apoe*$^{-/-}$ mouse (12-17 weeks) with intramural hematoma. The yellow arrow indicates the intramural hematoma. Scale bar = 2.5 mm.

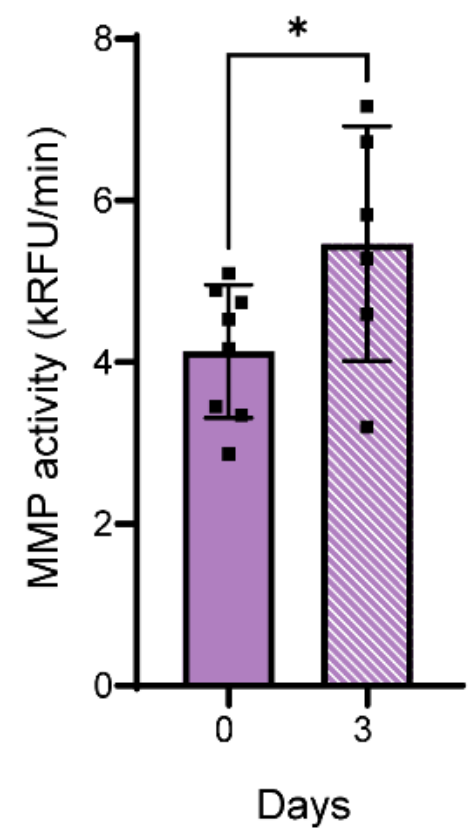

Supplemental Figure 9. Aorta MMP activity in control (day 0) and angiotensin II-infused *Apoe*$^{-/-}$ mice (12-17 weeks) at 3 days after starting angiotensin II infusion. **: $P < 0.01$. RFU: Relative fluorescence units.

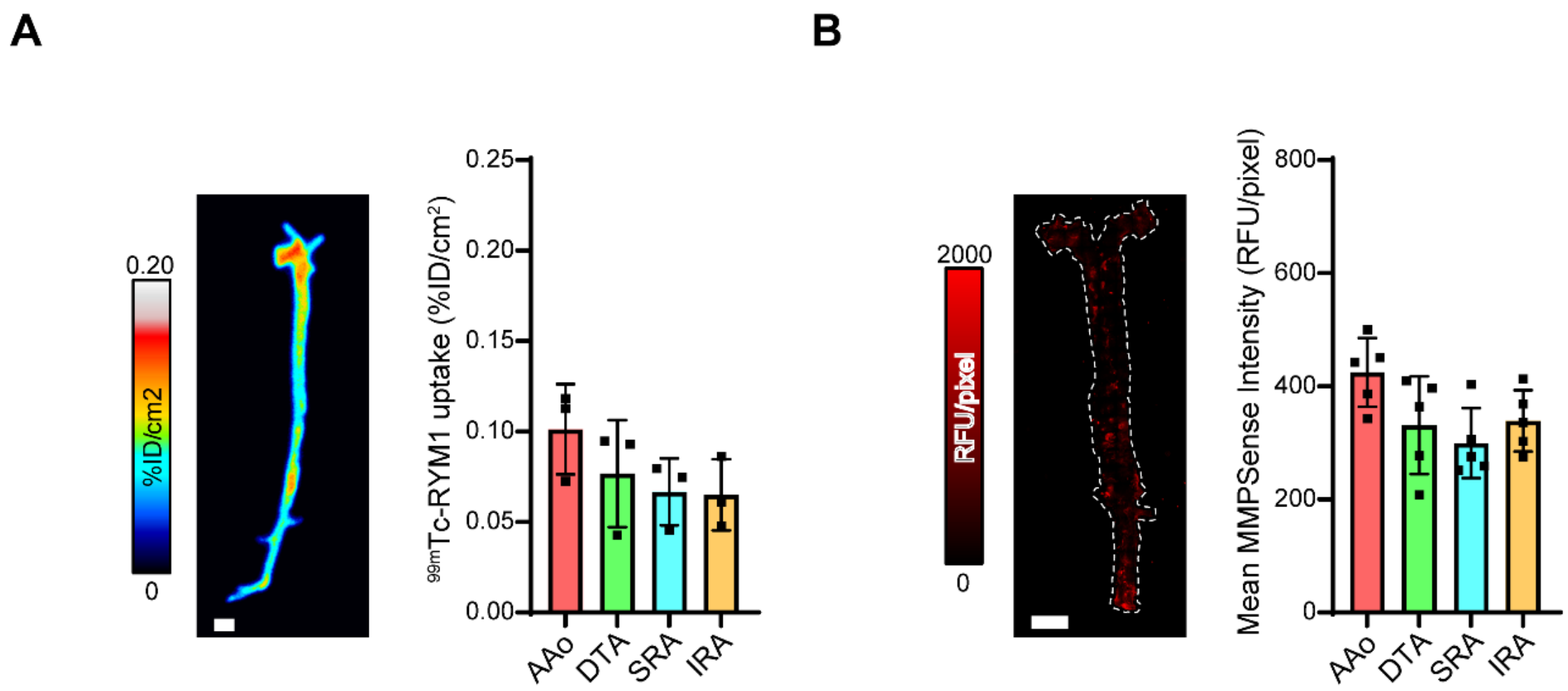

Supplemental Figure 10. Illustrative examples of ^{99m}Tc-RYM1 autoradiography (**A**) and MMPSense 645 fluorescence imaging (**B**) and their quantification in different segments of the aorta of 12-17 weeks *Apoe*$^{-/-}$ mouse. Scale bar = 2.5 mm. ID: injected dose, RFU: Relative fluorescence units, AAo: Ascending thoracic aorta, DTA: Descending thoracic aorta, SRA: suprarenal abdominal aorta, IRA: Infrarenal abdominal aorta.

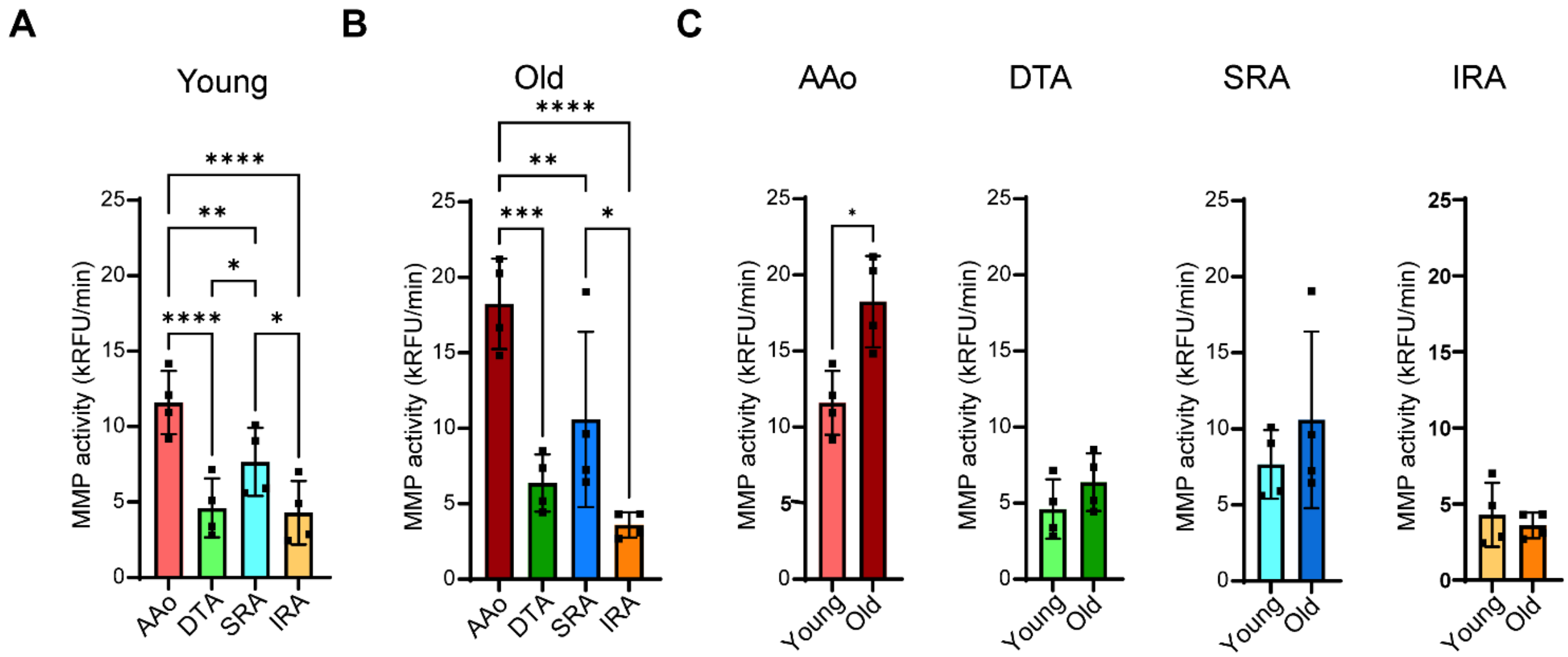

Supplemental Figure 11. MMP activity in different segments of the aorta of *Apoe*$^{-/-}$ mice. **A, B.** MMP activity in different aorta segments from young (8–10 weeks, **A**), and old mice (>51 weeks) mice (**B**). **C.** Comparison of MMP activity between young and old mice within each segment of the aorta. *: $P < 0.05$, **: $P < 0.01$, ***: $P < 0.001$, ****: $P < 0.0001$. RFU: Relative fluorescence units, AAo: Ascending thoracic aorta, DTA: Descending thoracic aorta, SRA: suprarenal abdominal aorta, IRA: Infrarenal abdominal aorta.

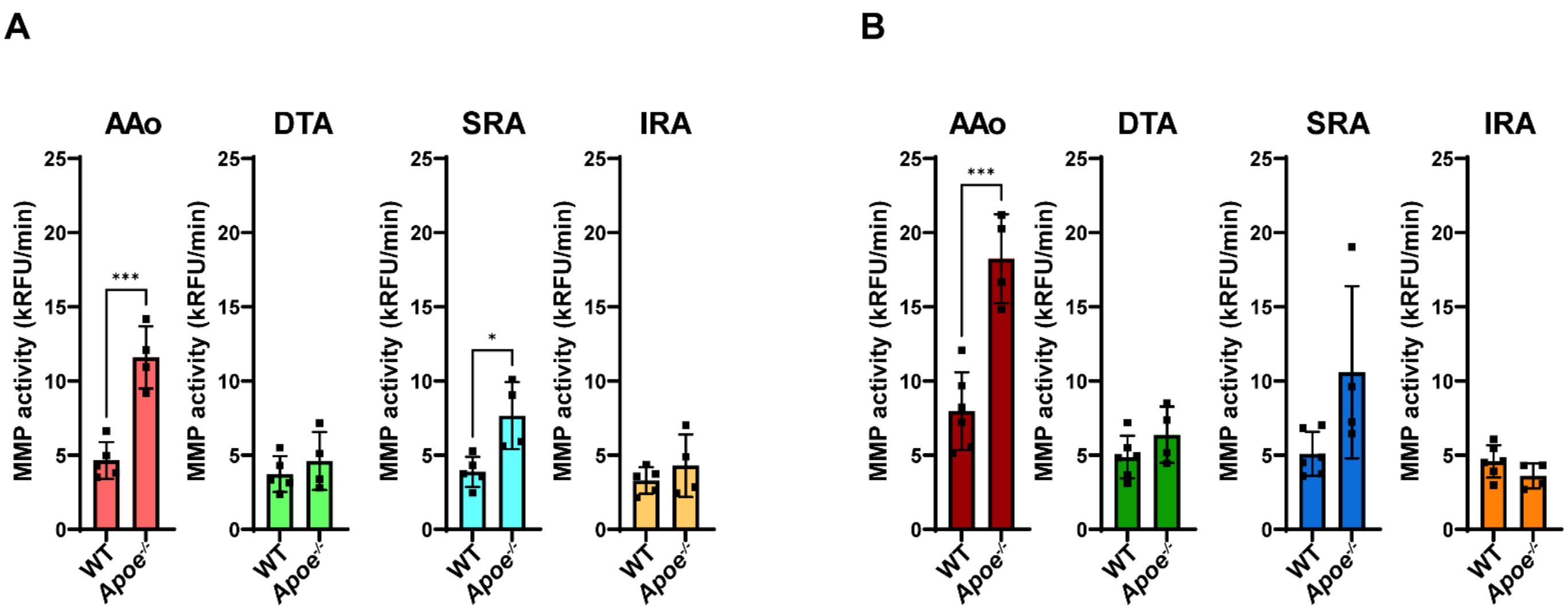

Supplemental Figure 12. Comparison of MMP activity in different segments of the aorta of young (8-10 weeks, **A**) and old (>51 weeks, **B**) wild type and *Apoe*$^{-/-}$ mice. *: $P < 0.05$, ***:$P < 0.001$. RFU: Relative fluorescence units, AAo: Ascending thoracic aorta, DTA: Descending thoracic aorta, SRA: suprarenal abdominal aorta, IRA: Infrarenal abdominal aorta.
